# Excited-State Dynamics of MAPbBr_3_: Coexistence of Excitons and Free Charge Carriers at Ultrafast Times

**DOI:** 10.1021/acs.jpcc.3c08509

**Published:** 2024-05-15

**Authors:** Nikolaos Droseros, Parnian Ferdowsi, Efrain Ochoa Martinez, Michael Saliba, Natalie Banerji, Demetra Tsokkou

**Affiliations:** † Department of Chemistry, Biochemistry and Pharmaceutical Sciences, University of Bern, Freiestrasse 3, Bern CH-3012, Switzerland; ‡ Adolphe Merkle Institute, Chemin des Verdiers 4, Fribourg CH-1700, Switzerland; § Helmholtz Young Investigator Group FRONTRUNNER, IEK5-Photovoltaics, Forschungszentrum Jülich, Jülich 52428, Germany; ∥ Institute for Photovoltaics, University of Stuttgart, Stuttgart 70569, Germany

## Abstract

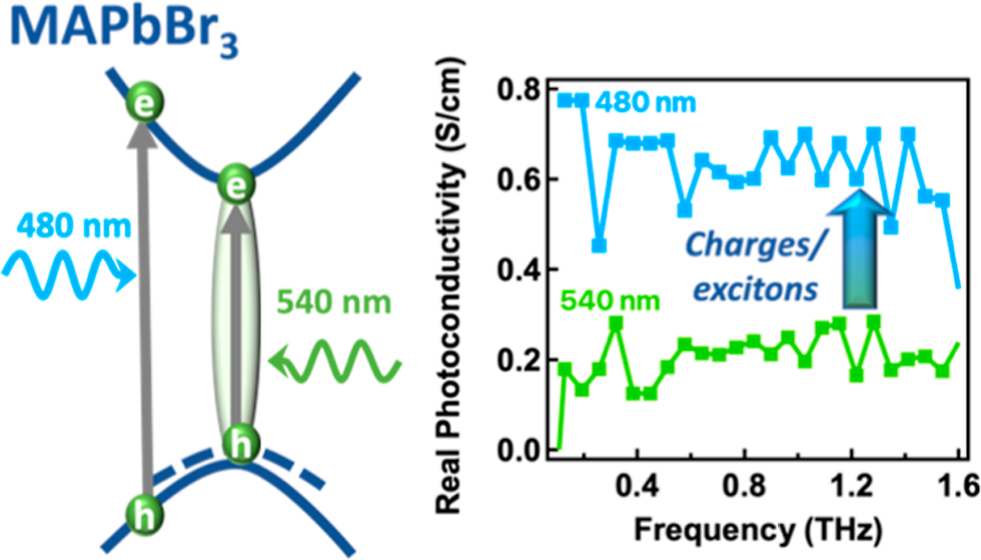

Methylammonium lead tribromide perovskite (MAPbBr_3_) is an important material, for example, for light-emitting applications and tandem solar cells. The relevant photophysical properties are governed by a plethora of phenomena resulting from the complex and relatively poorly understood interplay of excitons and free charge carriers in the excited state. In this study, we combine transient spectroscopies in the visible and terahertz range to investigate the presence and evolution of excitons and free charge carriers at ultrafast times upon excitation at various photon energies and densities. For above- and resonant band-gap excitation, we find that free charges and excitons coexist and that both are mainly promptly generated within our 50–100 fs experimental time resolution. However, the exciton-to-free charge ratio increases upon decreasing the phonon energy toward resonant band gap excitation. The free charge signatures dominate the transient absorption response for above-band-gap excitation and low excitation densities, masking the excitonic features. With resonant band gap excitation and low excitation densities, we find that although the exciton density increases, free charges remain. We show evidence that the excitons localize into shallow trap and/or Urbach tail states to form localized excitons (within tens of picoseconds) that subsequently get detrapped. Using high excitation densities, we demonstrate that many-body interactions become pronounced and effects such as the Moss–Burstein shift, band gap renormalization, excitonic repulsion, and the formation of Mahan excitons are evident. The coexistence of excitons and free charges that we demonstrate here for photoexcited MAPbBr_3_ at ultrafast time scales confirms the high potential of the material for both light-emitting diode and tandem solar cell applications.

## Introduction

Hybrid organic–inorganic perovskites with low exciton binding energy, high absorption coefficient, defect tolerance, a tunable band gap, high carrier mobility, and solution processability have emerged as efficient light harvesters for solar cell applications showing remarkable performance with efficiencies >26% on par with established single-crystalline silicon solar cells.^1−3^ On the other hand, perovskites with higher exciton binding energies and narrow emission bandwidth are promising candidates for perovskite light-emitting diodes (PeLEDs).^4−6^ Among the most studied representatives for PeLEDs is the green-emitting (at 2.3 eV) methylammonium lead tribromide (MAPbBr_3_), first used in 2014 due to its higher exciton binding energy compared to that of the iodide counterpart.^7−9^ For both applications, PeLEDs and solar cells, a deep understanding of the optical properties, the photogenerated species, and their recombination upon excitation is essential.

There has been extensive research on elucidating the origin of photoluminescence (PL) in MAPbBr_3_.^10−15^ Both free charge carriers^16^ and excitons^17^ participate in the emission of MAPbBr_3_, depending on the excitation density and crystal size. In nanocrystals, a smaller crystal size going down to the quantum confinement regime typically enhances the excitonic character and increases the photoluminescence quantum yield (PLQY).^11,12,18−21^ In polycrystalline MAPbBr_3_ with micrometer-size crystals, as obtained with standard solution-processing techniques, we have demonstrated the coexistence of free charges and excitons following photoexcitation on the nanosecond time scale.^11^ The ratio of the populations of free carriers to excitons depends on the excitation density.^11,12,21^ Highly emissive excitons are advantageous for PeLED applications,^21−23^ but the presence of free carriers is beneficial when MAPbBr_3_ is used as the high band gap material, e.g., in multijunction solar cells. Therefore, it is important to understand the interplay in the excited state also at very early times upon excitation (<1 ns). To access the contributions from charges and excitons, time-resolved photoluminescence (TRPL) has previously been employed. However, disentangling the contribution of the two species is not trivial, given the overlap of their spectral signatures and the difference in the oscillator strength of their transitions. This makes it challenging to directly translate a signal amplitude into the population of a specific species.^10,11^ The coexistence of excitons and free charge carriers results in different dynamics at the nanosecond scale between TRPL and time-resolved microwave conductivity (TRMC) measurements, the latter being selective to the free carriers.^13^ Most existing studies have focused on the slower time scales ranging from tens of nanoseconds to the steady state.^24^ Previous ultrafast studies have not been primarily concerned with disentangling the behavior, dynamics, and yield of the two species.^25−28^ Additionally, previous THz studies that isolate the behavior of free charges are scarce and tend to concentrate on the higher-order recombination processes occurring at high fluences or on the phonon modes at these frequencies.^29−32^

Here, we present an in-depth study of the photophysics of both excitons and free charge carriers in photoexcited MAPbBr_3_ on the ultrafast time scale (∼100 fs to 2 ns) by combining transient absorption (TA) and time-resolved terahertz (TRTS) spectroscopies. We achieve this by varying the relative population of the two species through above-band-gap vs resonant band gap excitation and by adjusting the excitation density. In TA measurements, the features of both species will overlap, while we selectively target the photoconductivity of free carriers on the picosecond time scale using TRTS. We show that both free charges and excitons coexist upon excitation, are promptly generated, and are long-lived. Because of the excess photon energy with the above band gap excitation, we observe a higher population of free carriers, which tend to mask the features of photogenerated excitons. At low pump fluences, the TA response is primarily dominated by the signatures of free charges, resulting in characteristic relaxation effects, such as band filling and the phonon bottleneck. In contrast, with resonant band gap excitation, fewer free carriers are generated, and the signatures of unscreened, directly photoexcited excitons become readily identifiable in the TA spectra. We find that photoexcited excitons are initially trapped in shallow trap distributions and/or localized to the Urbach tail states. However, at longer times, they become detrapped and coexist with free carriers until they eventually decay through band-to-band recombination. In this work, we demonstrate that excitation of high band gap MAPbBr_3_ at low excitation densities and excess excitation photon energy enhances the generation of free charges over excitons that are promptly generated (<100 fs), and have a long lifetime. This characteristic is advantageous, e.g., for designing absorbers in the blue part of the solar spectrum that are of interest for multijunction solar cells.

## Methods

### Sample Preparation and Film Characterization Methods

The MAPbBr_3_ film was deposited using the antisolvent technique.^33,34^ To get a film of ∼300 nm thickness, 40 μL of the perovskite precursor was spin-coated for 30 s at 3000 rpm. Ten seconds before the end of the spin coating, 100 μL of chlorobenzene was drop-cast on the film while spinning to start the film formation. To maintain the film safe from air exposure for the steady-state absorption measurements, 50 μL of PMMA with a concentration of 0.1 mgr/mL was dynamically spin-coated on top of the perovskite at 4000 rpm for 30 s. The film was subsequently annealed at 100 °C for 45 min. The complete film deposition process took place under a nitrogen (N_2_) environment inside a glovebox and films were not exposed to air before or during the spectroscopy measurements. The morphology of the perovskite film was studied via scanning electron microscopy (SEM) measurements performed using a TESCAN MIRA3 LM FE, by AZO MATERIALS. Steady-state absorbance spectra were performed using a LAMBDA 950 UV–vis–IR (PerkinElmer) spectrometer with the use of an integrating sphere.

### Time-Integrated and TRPL Spectroscopy

TRPL measurements were performed using excitation pulses generated by the fundamental near-UV pulses of a Q-switched Surelite Continuum Laser (3 ns, 355 nm, 10 Hz) and frequency converted in an optical parametric oscillator (GWU versaScan). For detection, an Andor iStar camera, coupled with a monochromator, was used. The time-dependent emission of the excited state was obtained by varying the time delay between the laser Q-switching and the camera detection time.

### Transient Absorption Spectroscopy

The excitation pulses were generated from the fundamental near-infrared pulses of an amplified Ti:sapphire laser system (35 fs, 800 nm, 1 kHz, 6 mJ, Astrella, Coherent) and frequency converted by an optical parametric amplifier (OPA, OPerA Solo, Coherent). Broadband white light probe pulses covering the visible and near-IR region from 450 to 1400 nm were generated in a 5 mm thick sapphire plate using part of the fundamental beam. The white light was split into two components that served as the signal and reference pulses. The probe pulses were temporally delayed relative to the excitation pulses via a micrometer translation stage, and pump–probe delays of up to 2 ns could be measured. Typically, pump pulses of ∼1 mm diameter were used to photoexcite the sample. The pump pulses were chopped at half the laser frequency before being spatially overlapped with the weaker probe pulses of ∼250 μm diameter on the sample. The different pump and probe beam diameters ensured a uniform distribution of detected photoexcited species. The role of the probe beam was to probe the changes induced by pump excitation at different pump–probe delays. The signal probe pulses transmitted through the sample and the reference probe pulses were spectrally dispersed in a home-built prism spectrograph assembled by Entwicklungsbüro Stresing, Berlin, and detected separately, shot-to-shot, by a pair of charge-coupled devices (CCD detectors, Hamamatsu S07030-0906). The recorded TA spectra were corrected for the chirp of the white light probe, taking into account the cross-phase modulation signal induced by high pump intensity on a glass substrate.

### Time-Resolved THz Spectroscopy

Time-resolved THz spectroscopy is an ultrafast pump–probe method in which visible pump pulses are used to photoexcite the sample similar to TA measurements, while the probe is a THz pulse, which has a small photon energy of a few meVs. In addition, a phase-sensitive detection technique allows the measurement of the THz electric field and not just its amplitude, unlike in TA spectroscopy. Thus, the complex photoconductivity spectra and photoconductivity dynamics are accessible with this method with a subpicosecond temporal resolution. Moreover, due to the short THz pulse duration, the motion of charge carriers over short distances on the order of nanometers is accessible. The measured THz photoconductivity is proportional to the product of the charge carrier density and its short-range mobility. On the contrary, TA spectroscopy is sensitive only to the dynamics of the photoexcited population. An additional difference between both methods is that since it accesses conductivity, THz spectroscopy is mainly sensitive to the detection of free carriers and not to that of neutral excitons.

THz pulses were generated via a second-order nonlinear effect named optical rectification when intense femtosecond pulses of 800 nm are focused in a nonlinear (110) ZnTe crystal. These pulses were then directed and focused on the sample via off-axis parabolic mirrors, where they noncollinearly overlapped with the visible pump pulses (generated at 480 or 540 nm in an OPA, OPerA Solo, Coherent). For the detection of the transmitted THz pulses, free-space electro-optic sampling was used. In order for the THz radiation transmitted through the sample to be detected, the transmitted pulses were focused again on a second (110) ZnTe crystal, where they overlapped temporally and spatially with a small part of the fundamental near-infrared laser beam that acted as a gate. The modification in the refractive index induced in the detector crystal by the propagation of the THz pulses caused a change in gate polarization that was used to obtain the time-domain profile of the THz electric field. The presence of moisture causes significant distortions in the THz spectrum since this radiation is strongly absorbed by water; therefore, all measurements were performed under continuous nitrogen flow. In this way, degradation of the perovskite film by moisture was also avoided. Two different kinds of measurements were performed. First, the photoconductivity dynamics at the maximum of the THz electric field were recorded by varying the pump–THz (probe) delay at a fixed delay between the THz and the gate pulses. Second, the photoinduced change of the transmitted THz electric field waveform with and without excitation of the sample was recorded at a fixed pump–THz delay time by scanning the time delay between the THz and the gate pulses. THz pulses have a duration of about 1 ps, limiting the temporal resolution of the experiments. Subsequently, these signals were used to extract the photoconductivity spectrum Δσ in the range between 0.1 and 1.6 THz using the appropriate analysis for thin films. The time delays between the pump–THz and THz–gate pulses were controlled independently by computer-controlled translation stages.

## Results and Discussion

### MAPbBr_3_ Film Characterization

**Figure 1:**
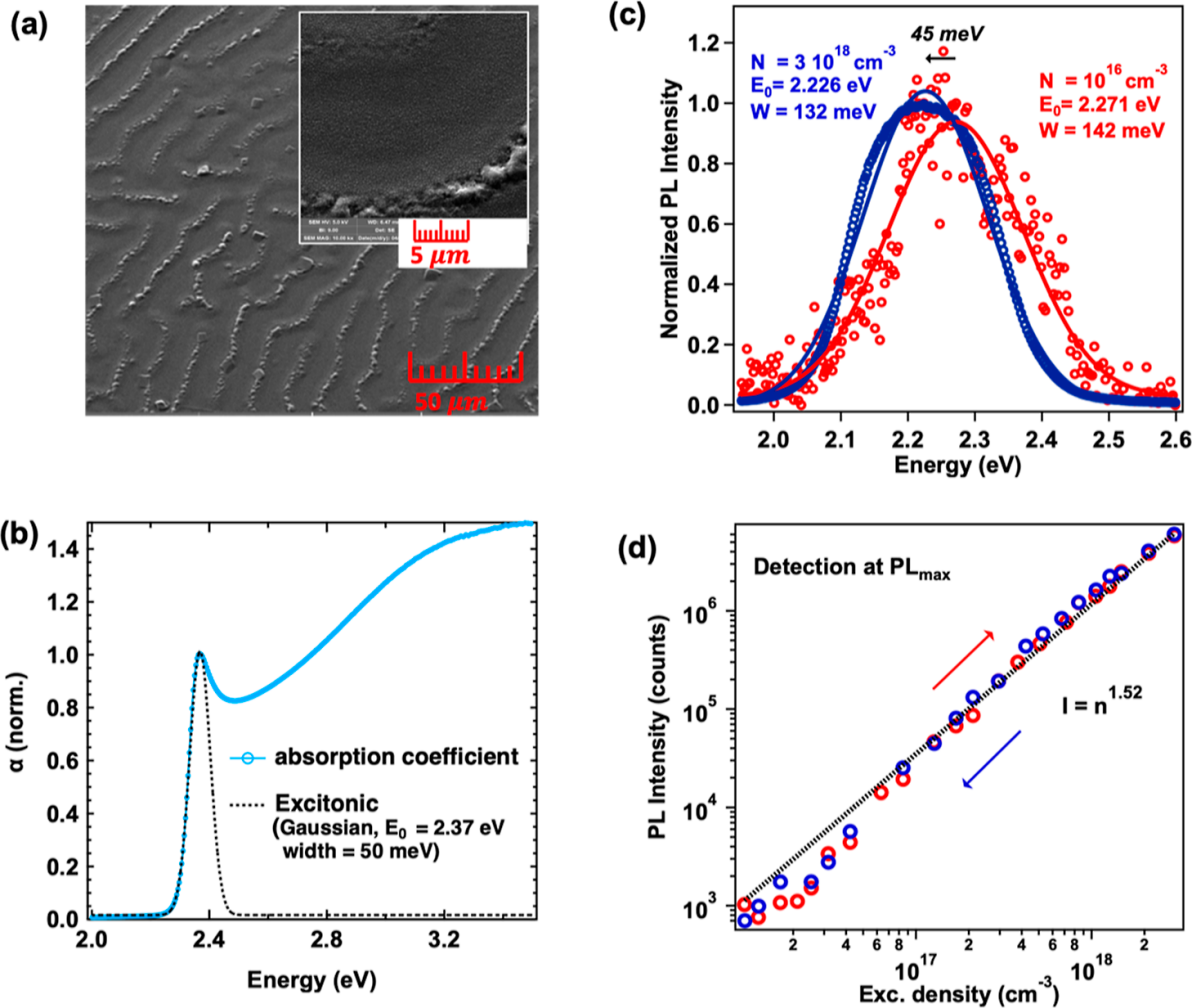
(a) SEM image of the MAPbBr_3_ film. The inset displays a higher magnification image. (b) Normalized absorption coefficient spectrum. The Gaussian analysis of the excitonic peak is included with a dotted line. (c) Time-integrated PL spectra obtained with low (10^16^ cm^–3^) and high (3 × 10^18^ cm^–3^) excitation densities. The Gaussian functions used for fitting the results are also depicted. Excitation at 450 nm was used. (d) Integrated PL intensity detected at the PL maximum, once with increasing (red circles) and once with decreasing (blue circles) excitation carrier density.

The morphology of the perovskite film, as studied via SEM images, is shown in Figure 1a and the inset. Two distinct morphologies are observed, one with wrinkles (as shown in Figure 1a),^35^ and flat regions between the wrinkles (as shown in the inset). Both regions consist of perovskite grains of the same size, approximately 300 nm. The formation of wrinkles is typical of the antisolvent deposition technique used in this study. However, we do not expect significant differences in our spectroscopic measurements, as the grain size is similar in both regions. This hypothesis is further supported by the absorbance spectrum shown in Figure 1b, which has been used to estimate the Urbach energy (*E*_u_ = 22 meV) (Figure S1, see Supporting Information Section S1). Such a low value is consistent with values reported in the literature, ranging between 17 and 23 meV,^36,37^ and indicates that the intrinsic electronic disorder close to the band edge is low, suggesting that the sample is quite homogeneous despite the presence of wrinkles. In the absorbance spectra (Figure 1b), the characteristic excitonic band of MAPbBr_3_ is present at 2.37 eV overlapping the continuum absorption at higher energies.

To obtain information about the nature of the species participating in the PL emission of MAPbBr_3_, the PL spectra obtained with low and high excitation densities are shown in Figure 1c. The increase in excitation density causes a red shift of the PL maximum by ∼45 meV and a spectral narrowing (Gaussian width from 142 to 132 meV). These observations are in agreement with the analysis performed in our previous study and are indicative of the coexistence of free carriers and excitons at low excitation densities and the conversion of free carriers into excitons at higher excitation densities.^11^Figure 1d shows the intensity detected at the PL maximum peak (2.23 eV) as a function of the excitation density. The lack of any hysteresis in the PL results confirms that the use of high carrier densities (up to 3 × 10^18^ cm^–3^) does not cause any permanent photodegradation for the investigated MAPbBr_3_ film. Further evidence of the coexistence of the two species and the film’s stability is provided by the integrated PL taken at different excitation densities, both increasing (red dots) and decreasing (blue dots) the carrier density (Figure S2a,b). The excitation density-dependent PL maximum peak (Figure 1d) was analyzed using a power-law function *n*^*k*^, where *I* is the PL intensity, *n* is the excitation density, and *k* is a real number exponent. This exponent offers insights into the order of the recombination process.^18,38,39^ From this analysis, a value of *k* = 1.52 was obtained, indicative of the coexistence of free carriers and excitons, consistent with previous studies.^11,17^ Additionally, by fitting the TRPL dynamics (Figure S2c), two recombination time constants were obtained, a faster one of τ_1_ = 2 ns and a slower one of τ_2_ = 15 ns, related to trap-assisted and band-to-band recombination, respectively.^11^ An average PL lifetime of 3 ns was calculated, similar to the value reported in our previous work at a low excitation density.^19^ However, these measurements provide information about the charges and excitons at long times (>1 ns); their behavior, coexistence, or interconversion at ultrafast times (<1 ns) is not accessed.

### Time-Resolved THz Measurements

**Figure 2:**
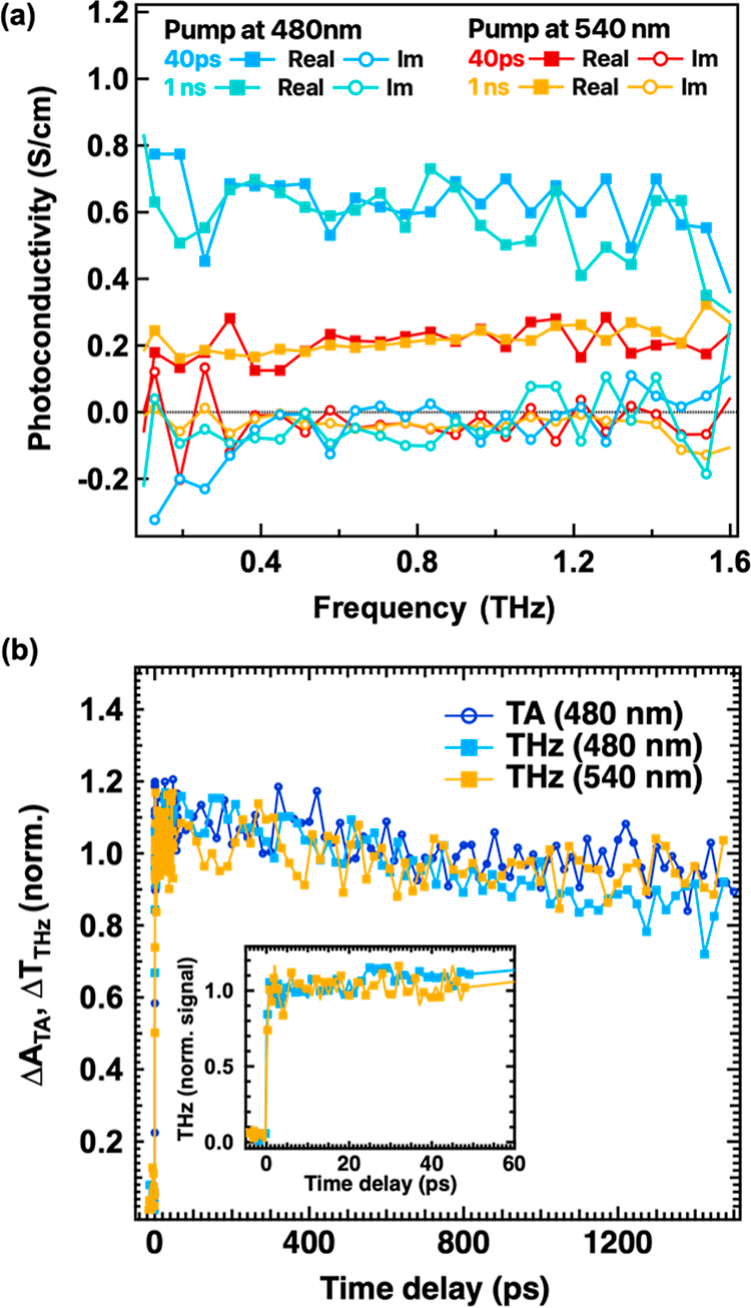
(a) Photoconductivity spectra recorded at different time delays after excitation at 480 and 540 nm with a photoexcited carrier density of 6.3 × 10^17^ cm^–3^, which is in the linear regime. Both real (squares) and imaginary (open circles) parts are displayed. (b) Normalized THz dynamics (Δ*T*_THz_) and TA (GSB) dynamics (Δ*A*_TA_) with 480 and 540 nm excitation. The THz dynamics at early times are included in the inset.

To isolate the behavior of free charges in MAPbBr_3_ at ultrafast times, we employ transient THz spectroscopy because excitons do not strongly interact with the THz radiation. We access the population of charged carriers by measuring the photoconductivity at two excitation wavelengths, when using above-band-gap excitation (480 nm) and resonant excitation of the excitonic peak (540 nm). Figure 2a displays the photoconductivity spectra Δσ recorded at two distinct time delays, 40 ps and 1 ns, for both excitations. For these measurements, we used a similar low excitation density of 6.3 × 10^17^ cm^–3^, where no higher-order recombination effects occur. The real parts (squares) and imaginary parts (circles) of the photoconductivity spectra are depicted. In all cases, we observe a positive nearly flat real part of conductivity, while the imaginary part is almost zero, indicating very weak charge carrier localization so that the probed charges are nearly free.^29,40^ Additionally, the absence of a strongly negative imaginary part rules out any contribution from excitons to the conductivity. We observe that at both excitations (480 and 540 nm), the spectra at early (40 ps) and late (1 ns) times exhibit similar shapes and amplitudes suggesting that the probed density of free carriers and their short-range mobility do not significantly change within this time window. The weak recombination/trapping of charges is also evident in the THz dynamics shown in Figure 2b. The main difference in the results is observed when comparing the two excitation wavelengths. For a similar absorbed carrier density, the amplitude of conductivity measured with 540 nm excitation is about three times smaller than that measured with 480 nm excitation. This discrepancy indicates a reduced number of free carriers, even at early times (∼1 ps) upon 540 nm excitation. Consequently, a higher density of excitons is generated by 540 nm excitation. This outcome aligns with expectations, as pump photons that excite carriers above the band edge at 480 nm can influence exciton photogeneration or formation due to the initial excess kinetic energy, leading to higher mobile charge yields. The fact that the maximum photoconductivity is recorded at early times (1 ps) suggests that charges are generated very early on, as further discussed below. Similar observations hold at 540 nm excitation, where the maximum conductivity is reached within the temporal resolution of these measurements. Comparable findings have been reported for MAPbI_3_ when using resonant excitonic excitation with 800 nm excitation.^41^ In those experiments, the faster time resolution (40 fs) revealed that charge carriers in MAPbI_3_ are formed due to exciton dissociation that takes place before 1 ps.^41^

In summary, the main conclusions drawn from the THz measurements are as follows: (i) free charges are generated at both excitations, (ii) there is a prompt generation of charges occurring within 1 ps, (iii) 540 nm excitation results in a higher population of excitons compared to 480 nm excitation, and (iv) there is no significant charge trapping or delayed exciton formation observed. These findings hold significant importance for interpreting the spectral changes and dynamics observed in the TA measurements for different photoexcitations. To directly investigate the ultrafast dynamics of both free carriers and excitons in MAPbBr_3_, the TA measurements were performed using the same excitation photon energies employed in THz measurements, to modify the ratio between the different photoexcited species. Further experimental details are given in the methods.

### Transient Absorption Spectroscopy with Above-Band-Gap Excitation

**Figure 3:**
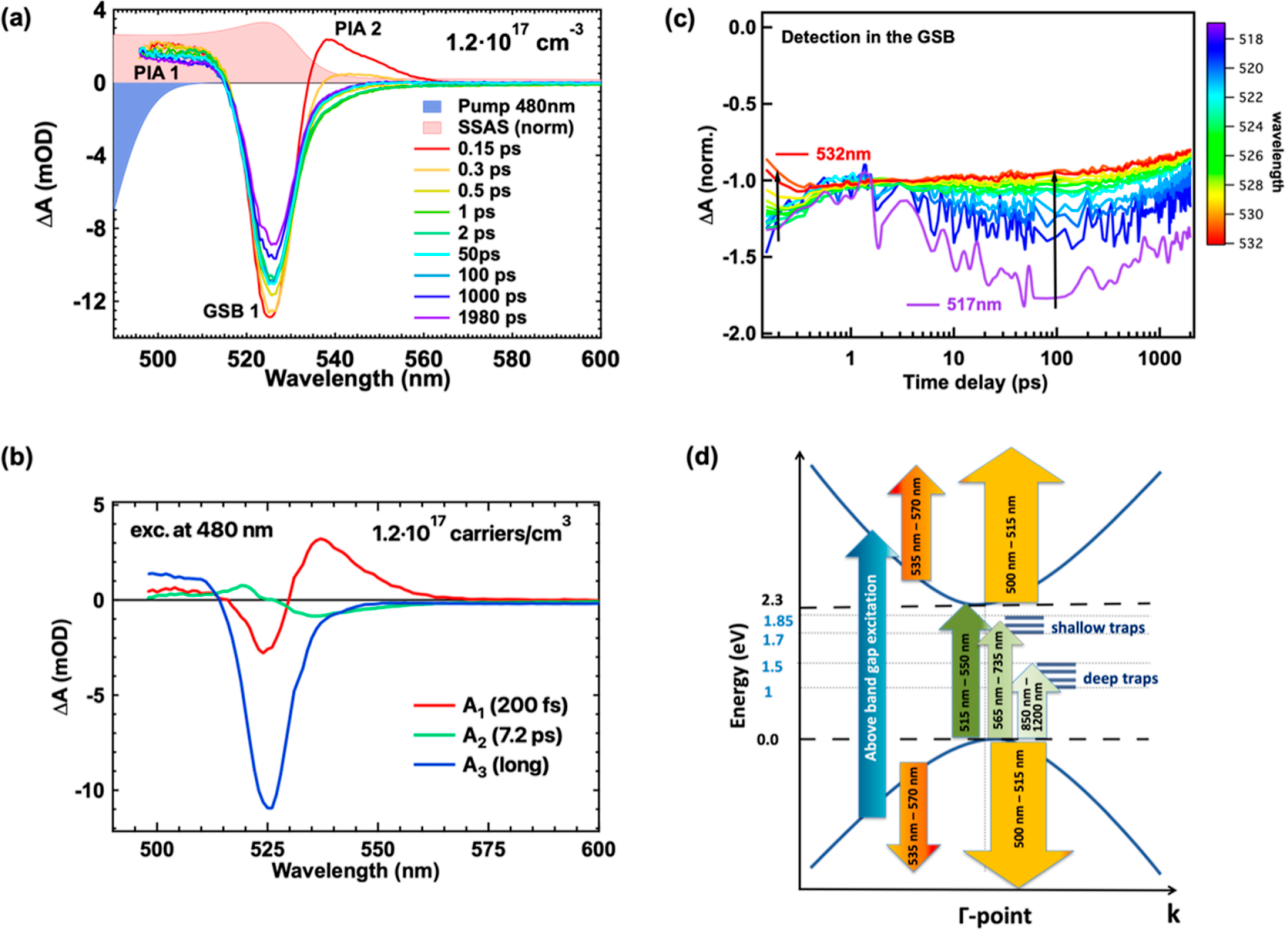
(a) TA spectra after excitation at 480 nm with a photogenerated carrier density of 1.2 × 10^17^ cm^–3^. The steady-state absorption spectrum (red) and the pump pulse width used (blue) are also shown, with their amplitude being arbitrary. (b) Decay-associated spectra of the MAPbBr_3_ film excited at 480 nm. (c) Normalized TA dynamics at 3 ps probed at different wavelengths in the GSB 1; the arrows point toward longer probed wavelengths. (d) Band diagram of the MAPbBr_3_ film around the Γ-point along with the detected optical transitions.

First, we discuss the results from TA spectroscopy for the above-band-gap excitation. In Figure 3a, we present the TA spectra of the MAPbBr_3_ film following excitation with 480 nm pulses at selected time delays after photoexcitation. Different spectral features are manifested in distinct spectral regions, namely, within the range of 500–515 nm, a positive signal (PIA 1) becomes evident. This signal arises from a combination of photoinduced absorption (PIA) and photoinduced changes in the refractive index due to generated free charges.^42^ Between 515 and 550 nm, a negative signal coinciding with the excitonic peak in the steady-state absorption spectrum is observed. This negative signal is attributed to ground-state bleaching (GSB 1), resulting from the phase-space filling of the excitonic transition caused by photoinduced species.^43,44^ As discussed in the context of iodide perovskites, the presence of free carriers with above-band-gap excitation masks the excitonic signatures due to a perfect cancellation between two opposite effects. These effects are (1) the reduction of the exciton binding energy due to screening by free carriers and (2) the reduction of the band gap energy due to the band gap renormalization (BGR) induced by the presence of electrons in the conduction band and holes in the valence band.^45−47^ Additionally, a second positive peak (PIA 2) emerges at early times spanning from 535 to 565 nm. This band rapidly converts into a negative extension of GSB 1 within 1 ps. Such rapid decay aligns with previous studies and is attributed to carrier cooling in MAPbBr_3_,^25^ as observed in iodide perovskites as well.^48,49^ At longer wavelengths (≥560 nm up to the near IR), a weak negative signal predominates (Figure S3a), which we ascribe to the presence of trap states. In this range, we distinguish two distinct trap distributions: shallow traps (ranging from 565 to 660 nm) and deep traps (ranging from 830 to 1150 nm), because these negative bands, denoted as BL1 and BL2, evolve differently with time (Figure S3b).

With 480 nm excitation, the features and dynamics correspond to those expected from the free charges. This is further justified by the similar GSB 1 and photoconductivity dynamics (Figure 2b), revealing no significant change in the charge local mobility and little charge density decay up to 1.8 ns. This denotes that (i) for times slower than 1 ps (which is the temporal resolution of THz measurements), the TA features/dynamics for above-band-gap excitation are governed by the free charges, (ii) charges are present at times as fast as 1 ps and no significant slow dissociation of excitons is seen at longer times, and (iii) charges weakly recombine within the time window of the measurement. Therefore, any potential conversion of charges to excitons is already completed during the first 1 ps after photoexcitation. Additionally, the THz signal remains constant between 1 and 40 ps (inset of Figure 2b), thus showing weak trapping in agreement with the small amplitude of GSB for the shallow and deep trap states.

To elucidate the mechanisms governing the dynamics in MAPbBr_3_ and to track their contribution to various spectral regions, a global fitting analysis was employed for our TA results (Figure 3b). In this procedure, the entire TA data set was simultaneously fit to determine the relaxation mechanisms and the corresponding time constants associated with each process. The data were simulated by summing exponential decays convoluted with an instrument response Gaussian function, where *A*_*i*_ represents the amplitude, and τ_*i*_ represents the corresponding time constant for each relaxation mechanism. Each time constant is assigned to a relaxation/recombination process, and the amplitude corresponds to the portion of the TA signal involved in that specific process. Figure 3b displays the decay-associated amplitude spectra obtained by fitting the data following 480 nm excitation.

For 480 nm excitation, the fast component with a time constant of 200 fs corresponds to the charge carrier cooling. During this process, carriers from higher energy states relax toward the band edge, resulting in the decay of the blue part of the GSB (higher energy states) and the rise of the red part corresponding to band edge states. This effect is clearly illustrated when examining the normalized GSB dynamics shown in Figure 3c, where a fast decay is obtained at shorter wavelengths (517 nm) followed by a slower rise at longer wavelengths (532 nm), at <3 ps.^50^ The data were normalized at 3 ps to facilitate guidance for the eye. The observation that PIA 2 decays with the same time constant indicates that this band arises from the absorption of higher energy states.

The intermediate time constant of 7.2 ps includes a positive amplitude at short wavelengths and a negative amplitude at long wavelengths within the GSB 1 band. This behavior becomes even more evident when examining the GSB 1 dynamics for times >3 ps, as depicted in Figure 3c. As indicated by the arrow pointing toward longer detection wavelengths, the dynamics at shorter wavelengths (corresponding to higher energy levels) exhibit a slower rise time extending up to 20 ps in contrast to the dynamics observed at longer wavelengths, which are closer to the band edge. This is associated with photoexcited charge carriers that lose their excess energy through the emission of longitudinal optical phonons, resulting in the formation of a large density of phonons.^42^ This leads to reabsorption of optical phonons by the charges, causing them to repopulate the higher excited states. Consequently, the relaxation of charge carriers located at the band edge slows down, an effect which is known as the phonon bottleneck.^43,50,51^ This effect slows the dynamics of the carriers at the higher states in comparison to those closer to the band edge, giving rise to a positive amplitude A_2_ at higher energies within the GSB 1 and a negative amplitude at lower energy states. We rule out that the intermediate time constant is related to BGR since this should be followed by a decay of charges which is not seen in the THz dynamics at the respective time delays.^43^ Additionally, trapping at the shallow trap states and deep trap states (resulting in BL1 and BL2, respectively, as shown in Figure S3a) may contribute as negative amplitudes.^25,26,52^ Shallow trap states capture the electrons from states closer to the band edge, effectively depopulating the band edge with a time constant that competes with the phonon bottleneck. At shorter wavelengths, the phonon bottleneck has a more pronounced impact on carrier dynamics, leading to a slow rise, as evidenced by the positive amplitude A_2_. The time constant extracted for the phonon bottleneck aligns with the lifetime of the C–N symmetrical stretch phonon mode (2 ps at room temperature), as observed in a THz study by Leguy et al.,^53^ providing support to our interpretation.

Finally, the mechanism represented by the amplitude A_3_ corresponds to a long time constant that exceeds our time window and is attributed to band-to-band and trapping recombination occurring at longer times, as seen in our TPRL measurements. By combining the results obtained for the optical band gap E_g_ (Figure S1) and the TA measurements, we construct a band diagram for MAPbBr_3_ around the Γ-point, as shown in Figure 3d. This diagram includes the energies associated with the shallow and deep trap states, along with the detected inter- and intraband optical transitions [PIA 1 (light orange), PIA 2 (dark orange), GSB 1 (dark green), BL 1 (green), and BL 2 (light green)]. As the PIA 2 band is related to carrier cooling, it involves higher energy states within the conduction and valence bands. The PIA 1 band is long-lived and has a weak early decay showing that both higher conduction/valence band energy states and band edge states contribute.

Our findings indicate that the TA spectra obtained with 480 nm excitation are primarily influenced by the signatures of free carriers. This is evident from the absence of distinctive narrow excitonic bands, the presence of carrier cooling, and phonon-assisted relaxation. Moreover, we do not see any evidence for conversion of charges to excitons or vice versa within the times measured, showing that the species are promptly photogenerated upon the above-band-gap excitation. These observations stimulated further investigation into the behavior of the MAPbBr_3_ film when exciting a reduced ratio of free carriers compared to excitons, to reveal the excitonic features in the TA spectra. To achieve a reduced ratio of free carriers over excitons, we used 500 and 540 nm excitations. In the case of 500 nm excitation, we obtained similar results to those with 480 nm excitation, although the excitation wavelength was tuned closer to the band edge (Figure S4). In this case, free carriers also dominate the spectral signatures.

### Transient Absorption Spectroscopy with Resonant Excitonic Excitation

**Figure 4:**
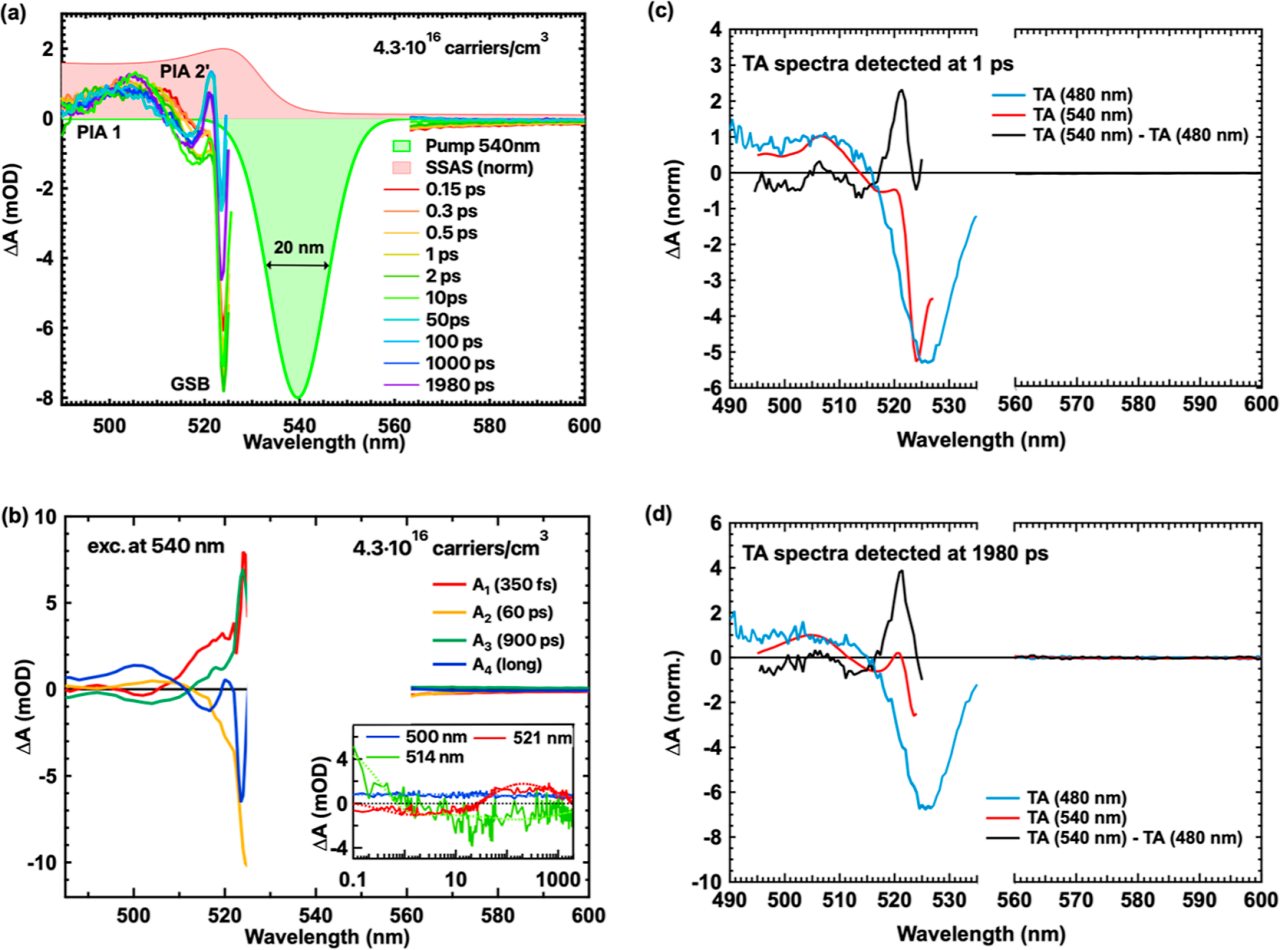
(a) TA spectra following excitation with 540 nm at selected times and a photoexcited carrier density of 4.3 × 10^16^ cm^–3^. The steady-state absorption spectrum (red) and the pump pulse width used (green) are also shown, with their amplitude being arbitrary. (b) Decay-associated spectra of the MAPbBr_3_ film excited at 540 nm. The inset in Figure 4b displays TA dynamics at various probed wavelengths along with their respective fits derived from the multiexponential global analysis. (c,d) TA spectra with 480 nm (blue line) and 540 nm (red line) excitations and their difference spectra (black line) detected at (c) 1 and (d) 1980 ps. Prior to subtraction, the TA spectra for both excitations were normalized at 505 nm.

So far, our studies of 480 and 500 nm excitation have primarily elucidated the role of free carriers, effectively concealing the excitonic signatures in the TA spectra. However, considering our previous study on the impact of excitons on recombination at later times (>1 ns),^11^ it becomes crucial to investigate their early time dynamics, which can provide essential parameters like the time constant for their formation and their interactions at different excitation densities. As evident from our THz measurements, this investigation can be effectively accomplished through 540 nm excitation resonant with the excitonic transition. Under this condition, the density of excitons is notably higher than that obtained through the above-band-gap excitation. In Figure 4a, we present TA spectra at various time delays following excitation with 540 nm at an excitation density of 4.3 × 10^16^ cm^–3^.

The TA spectra obtained with 540 nm excitation exhibit significantly different spectral signatures compared to the ones recorded with 480 and 500 nm excitations because of the increased density of photogenerated excitons. Notably, a GSB band is present coinciding with the excitonic absorption band, and it is much narrower than the corresponding band with 480 and 500 nm excitations. The sharpening of the GSB is partly attributed to its overlap with the additional PIAs, as discussed below. A first PIA 1 band ranging from 480 to 510 nm closely resembles the one obtained via 480 nm excitation, and it is associated with free charges. This band is observed from early times (as indicated in the inset of Figure 4b) and remains relatively the same up to 1 ns, suggesting that both charges and excitons are promptly generated. This is further supported by the similar THz conductivities for 540 nm excitation at early and late times (Figure 2a,b). Moreover, the charges exhibit long lifetimes, as indicated by PIA 1 and the THz dynamics (inset of Figures 4b and 2b), which do not significantly decay within 1 ns. A narrow second band (PIA 2′) from 520 to 525 nm becomes apparent only at times longer than 60 ps (inset of Figure 4b). This PIA 2′ band, positioned at energies just above the GSB, is a characteristic spectral signature of excitons, as supported by previous research.^54,55^ Furthermore, the presence of two trap distributions is also evident at longer wavelengths, as shown in Figure S5a.

Global analysis was applied to the data shown in Figure 4a, and the results are shown in Figure 4b. The amplitude spectra and associated time constants differ noticeably from those obtained with 480 nm excitation, primarily due to the presence of the excitons. In this case, both excitons and free carriers are mainly promptly generated and the short time constant of τ_1_ (=350 fs) corresponds to a fast relaxation of the excitons. The subtle rise of the 505 nm band in the fast component (negative amplitude) is attributed to charges and indicates weak exciton dissociation to charges at these short times (as shown in Figure 4b). Considering that charges do not show significant decay within 1 ns upon 540 nm excitation, as shown in THz dynamics, we conclude that any observed changes in the TA dynamics after the first 1 ps predominantly reflect the dynamics of the excitons that have promptly generated upon photoexcitation. We display the TA dynamics at various wavelengths in the inset of Figure 4b. The TA dynamics at 514 nm exhibit a very fast decay and the 521 nm signal (PIA 2′) changes to positive, leading to the observation of the narrow excitonic band (>10 ps). Since the population of free carriers remains constant, as indicated by the TA dynamics at 500 nm (PIA 1 and THz dynamics), any decaying signal should be attributed to either a reduction in the population or the relaxation of excitons.

To better understand the origin and the associated spectra of the longer time constants (with A_2_, A_3_, and A_4_ amplitudes) obtained from global analysis, first, we isolate the TA spectra associated with excitons by removing the signatures originating from the free carriers upon 540 nm excitation. To accomplish this, we subtracted the TA spectrum obtained with 480 nm excitation from the TA spectrum obtained with 540 nm excitation. The results at both early (1 ps) and later (1980 ps) times are presented in Figure 4c,d, respectively. Prior to the subtraction process, the TA spectra for both excitations were normalized at 505 nm, since this band primarily arises from photogenerated free charges. The remaining signal associated with free carriers upon excitation with 540 nm is effectively subtracted, revealing a positive band at 521 nm, corresponding to the positive excitonic peak (PIA 2′) observed at long times in Figure 4a. This narrow positive band arises from the broadening of the excitonic transition and has been previously observed in 2D MAPbI_3_^56^ and upon resonance excitation of 3D MAPbI_3_.^49,57^

The associated spectra related to the slower time constants contain the characteristic peaks from excitons, confirming that these are the species involved. The second decay mechanism with a time constant of 60 ps likely arises from a combination of exciton trapping to defects in the material close to the band edge and to the localization of free excitons to the tail states of the Urbach tail, which is an exponential tail of the density of states resulting from intrinsic disorder.^17^ At even longer times, on the order of hundreds of ps, GSB 1 exhibits a rise again with a time constant of τ_3_ = 900 ps and the associated spectrum has an antisymmetric shape to the one of A_2_. Therefore, we attribute this to the detrapping of excitons. Lastly, the long-lived signal, characterized by the A_4_ amplitude spectrum, represents the coexistence of free carriers and localized excitons at long times. Eventually, these species undergo band-to-band recombination outside of the TA temporal window. Following resonant excitation, we demonstrate the coexistence of excitons and charges, but the higher yield of excitons allows us to unravel their signatures and resolve their dynamics. We also show that both species are mainly promptly generated.

### Carrier Density-Dependent Transient Absorption Measurements

**Figure 5:**
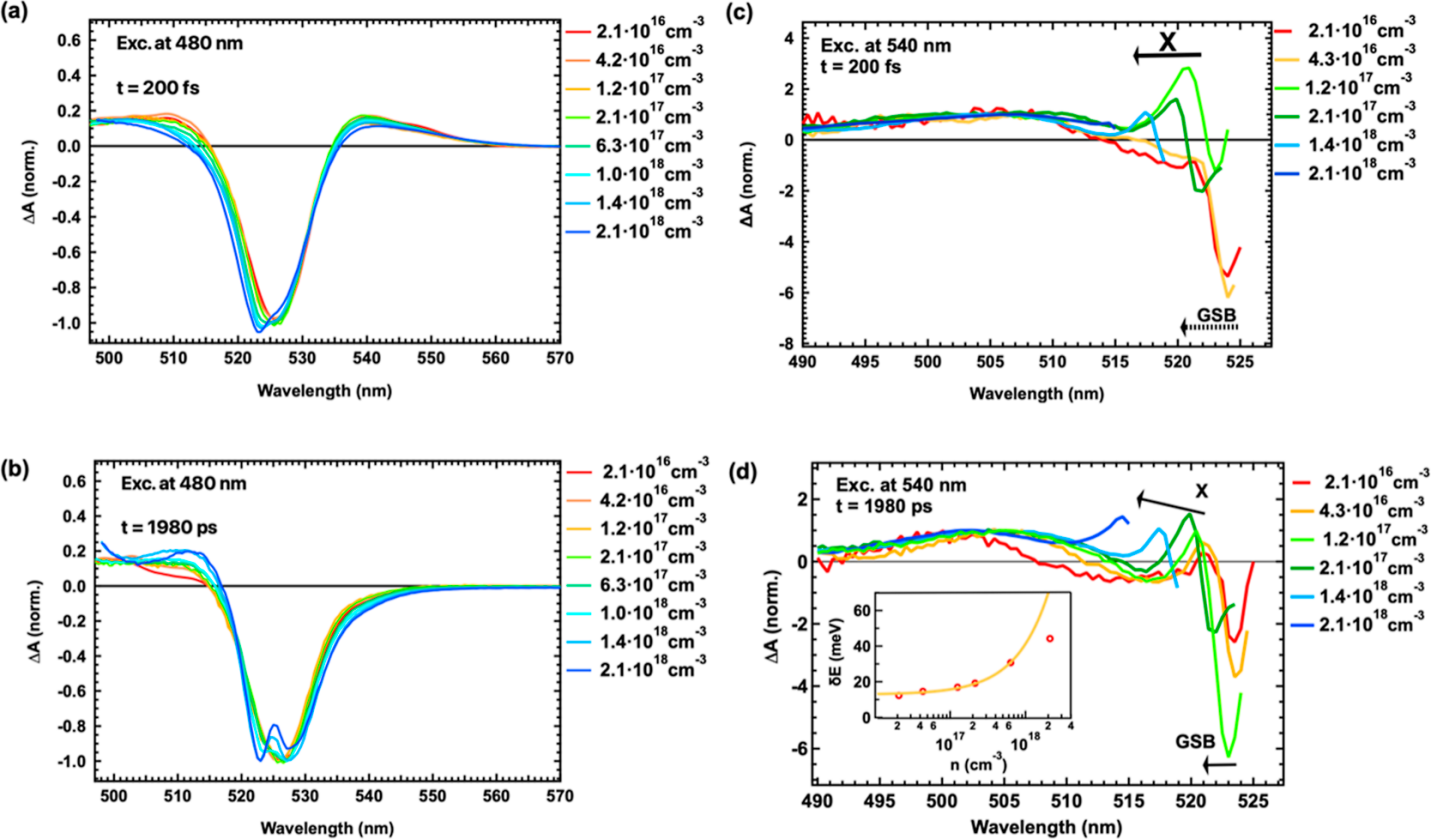
TA spectra under various excitation densities with 480 nm excitation detected (a) at 200 fs and (b) at 1980 ps after excitation. TA spectra under various excitation densities with 540 nm excitation, detected (c) at 200 fs and (d) at 1980 ps after excitation. The inset in (d) displays the energy difference between the positive excitonic peak and that in the steady-state absorption spectrum. The analysis of the data with a linear function is included in the same graph. Here, a logarithmic scale is used for the *x*-axis.

To gain a deeper insight into the many-body interactions and the species excited at high carrier densities, we performed TA measurements at various excitation densities for both 480 and 540 nm excitation. The TA spectra at different excitation densities and selected time delays after 480 and 540 nm excitation are shown in Figures S6a–g and S7a–e. Figure 5a,b shows the normalized TA spectra under different excitation densities with 480 nm excitation at early (200 fs) and long (1980 ps) times, respectively. Changes in the TA spectra occur for higher excitation densities. The changes in the early time TA spectra for above-band-gap excitation (Figure 5a) result from many-body interactions because of increased band filling leading to BGR and a Moss–Burstein shift.^28,43,50^ The latter is reflected in a blue-shifted and broader GSB band at early times as the excitation density is increased. Moreover, the disappearance of the PIA 2 band at late times enables the observation of long-lived BGR, manifesting as a red shift of the GSB with carrier density, due to the reduction in the band gap with increasing carrier density.^43^ Additionally, Figure 5a reveals a decrease in the amplitude of the PIA 2 band with increasing carrier density, which could be attributed to reduced carrier cooling toward the band edge. This could be due to higher-order effects or enhanced exciton generation upon photoexcitation. At late times and high excitation densities, as seen in Figure 5b, a narrowing of the PIA 1 band occurs, indicative of enhanced excitonic absorption. At high fluences, as the population of excited free carriers decreases with time, reduced screening of the excitons is expected. Consequently, an increase in the exciton oscillator strength results in enhanced exciton absorption. The rise in exciton yield at higher fluences leads to the splitting of the GSB into two bands, one at 523 nm and the other at 528 nm, at high excitation density.

Figure 5c,d displays the normalized TA spectra for 540 nm excitation at 200 fs and 1980 ps, respectively. The data were normalized at the peak of the PIA 1 band, which is primarily associated with free charges. However, the spectra are obscured by scattering caused by the high-intensity pump pulses, which is why the entire spectral range is not displayed. In Figure 5c, the excitonic peak PIA 2′ becomes apparent at very early times when the excitation density increases above 4.3 × 10^16^ cm^–3^, indicating an increase in the density of excitons compared to free charges. The presence of the PIA′2 band at later times demonstrates that excitons are present throughout the entire time window of the measurement and at all fluences. Moreover, as the excitation density increases, it leads to a blue shift of both PIA 2′ and GSB, observed at both early and late times. The inset of Figure 5d displays the energy difference between the PIA 2′ excitonic peak and the excitonic peak in the steady-state absorbance spectrum, which was obtained by fitting the latter with a Gaussian function (see Figure 1c). In the same graph, there is a linear fit of the energy shift with the carrier density. This shows that the energy shift increases linearly with the excitation density until it reaches an excitation density of 6.3 × 10^17^ cm^–3^. Several factors can contribute when high pump fluences are used: first, an optical Stark effect caused by the electric field of the pump;^58−60^ second, the screening of the excitons due to the long-lived free carriers;^46,54,55,61,62^ and third, the repulsion between excitons.^46,54,55,61,62^ We rule out the optical Stark effect, as it is expected to occur only at ultrafast times, primarily during the pump duration,^58,59^ while the persistent blue shift of the excitonic peak is observed even at long times (Figure 5d). Regarding the exciton screening due to the presence of the free carriers, it is expected to only affect the amplitude, but cannot account for the blue shift of the excitonic peak.^63^ Therefore, the blue shift of the excitonic peak and the GSB band with an increasing excitation density can be attributed to the repulsion between excitons. The repulsion occurs due to the Fermi nature of the constituents, namely, the electrons and the holes, which obey the Pauli exclusion principle.^45^

With 540 nm excitation, more excitons are generated compared to the 480 nm excitation and the short-range repulsion between the excitons is not compensated by an equivalent red shift from screening effects.^45,64^ Since the repulsion between excitons results in a positive potential energy greater than the energy of an isolated exciton, the energy cost for creating extra excitons in the system increases, leading to the blue shift of the excitonic peak with increasing excitation density.^46^ According to Litvinenko et al.,^65^ the blue shift of the exciton resonance energy has a linear dependence on the excitation density, because this leads to the generation of more excitons. The excitonic repulsion and the increase in exciton density also provide an explanation of the splitting of the GSB band at both short and long times upon 480 nm excitation (Figure 5a,b). At short times and low excitation densities, the excitons are efficiently screened by free carriers. As the excitation density increases, more free carriers are converted into excitons but the presence of a large population of free carriers still masks the excitonic peak. Only at the highest fluence does the excitonic peak become distinct. The deviation of δ*E* from the linear behavior at the highest excitation density of 2.1 × 10^18^ cm^–3^ can be attributed to nonlinear interactions among the photogenerated excitons, including Mahan excitons.^66^ Such a high excitation density is similar to the Mott density,^66−68^ where the excitons are expected to split into electrons and holes.^69^ Mahan demonstrated the existence of excitons even at high photoexcited densities, and this has been recently experimentally proven for MAPbBr_3_ associated with its relatively high exciton binding energy.^69^ For 540 nm excitation, an increase in the amplitude of the excitonic PIA 2′ peak compared to PIA 1 is observed up to the excitation density of 1.2 × 10^17^ cm^–3^. However, at even higher excitation densities, the opposite behavior is observed, where the spectral weight is transferred from the exciton peak PIA 2′ to the PIA 1. Lastly, there is an increase of the PIA 2′ peak at the excitation density of 2.1 × 10^18^ cm^–3^ (Figure S5f), which is also consistent with the presence of Mahan excitons.

## Conclusions

In conclusion, we conducted an in-depth investigation into the ultrafast dynamics of MAPbBr_3_ to unravel the behavior of both free charges and excitons. By combining various time-resolved spectroscopies, we find that MAPbBr_3_ excitation leads to the generation and coexistence of charges and excitons, and we vary their ratio by changing the excitation photon energy. We gained insights into various mechanisms and effects that take place at ultrafast times following photoexcitation, including band filling, BGR, phonon bottleneck, and trapping to shallow and deep trap states. From these, we constructed the band diagram of the MAPbBr_3_ perovskite, including the probed transitions via TA spectroscopy. We show an increase in the density of photogenerated excitons upon resonant excitation and we were able to uncover the ultrafast dynamics of excitons, which are typically obscured when above-band-gap excitation is used. Many body interactions are evident at high excitation densities arising from the repulsion between excitons and the presence of Mahan excitons. In summary, our study demonstrates the coexistence of both charges and excitons that exhibit prompt generation (within 100 fs) and long lifetimes (>1 ns). This behavior distinguishes MAPbBr_3_ from lower band gap perovskite materials, such as iodide and mixed iodide–bromide variants, where free charges play a dominant role. These findings underscore the potential of MAPbBr_3_ as a high-bandwidth material with promising applications in both PeLEDs and solar cells.

## Data Availability

The data that support the findings of this work are available as open accessin the BORIS Repository of the University of Bern at https://doi.org/10.48620/396.

## Supporting Information Available

Calculation of the Urbach energy; fluorescence emission in MAPbBr_3_; trap states probed with 480 nm excitation; transient absorption spectra with 500 nm excitation; trap states probed with 540 nm excitation; transient absorption spectra with 480 nm excitation at different densities; and transient absorption spectra with 540 nm excitation at different densities (PDF)
