# Supplementary material for: Excited-State Dynamics of MAPbBr3: Coexistence of Excitons and Free Charge Carriers at Ultrafast Times
Source: J Phys Chem C Nanomater Interfaces. 2024 May 15;128(21):8637–48. doi: 10.1021/acs.jpcc.3c08509 (PMC11145650; doi:10.1021/acs.jpcc.3c08509)
Supplement: Supplementary file 1 — jp3c08509_si_001.pdf [file jp3c08509_si_001.pdf]

Supporting Information

Excited-State Dynamics of $MAPbBr_3$: Coexistence of Excitons and Free Charge Carriers at Ultrafast Times

Nikolaos Droseros,[1] Parnian Ferdowsi,[2] Efrain Ochoa Martinez,[2] Michael Saliba,[3,4] Natalie Banerji*,[1], Demetra Tsokkou*[1]

[1] Department of Chemistry, Biochemistry and Pharmaceutical Sciences, University of Bern, Freiestrasse 3, CH-3012 Bern, Switzerland

[2] Adolphe Merkle Institute, Chemin des Verdiers 4, CH-1700 Fribourg Switzerland

[3] Helmholtz Young Investigator Group FRONTRUNNER, IEK5-Photovoltaics, Forschungszentrum Jülich, Jülich, Germany

[4] Institute for Photovoltaics, University of Stuttgart, 70569, Stuttgart, Germany

S1. Calculation of the Urbach energy

Figure S1 (a) shows the measured transmittance, reflectance and the calculated absorptance. The correction of the data derived from the steady state absorption measurement is necessary to remove artefacts and calculate accurately the absorption coefficient. The absorption coefficient α was calculated using the following equation: [1]

$$\alpha = -\frac{1}{t} \cdot \ln\left(\frac{T\%}{100 - R\%}\right) \tag{1}$$

where t is the film thickness. The normalized absorption coefficient, α, at the excitonic peak is shown in Figure 1 (c). The excitonic peak is located at 2.368 eV and has a width of 50 meV (11 nm) as extracted by analysis with a gaussian function also included in the figure.

Figure S1 (b) shows the Tauc plot for the studied perovskite. For a direct bandgap semiconductor, dependence of the absorption coefficient α on the photon energy E is:

$$(ahv)^2 \sim E \tag{2}$$

Fitting a linear equation to the linear part of the curve (Figure S1 (b)) and extrapolating this line to intercept the x- axis gives the optical bandgap of this perovskite, which equals 2.31 eV.

Figure S1 (c) shows the calculation of the Urbach energy of the perovskite that represents the disorder present close to the bandgap. The Urbach energy is given by the equation: [1], [2]

$$\alpha = Ae^{\frac{hv}{E_u}} \tag{3}$$

where, α is the absorption coefficient, h is Planck's constant, v is the frequency of the detected light, A is a pre-exponential factor and E_u the Urbach energy.

(a)

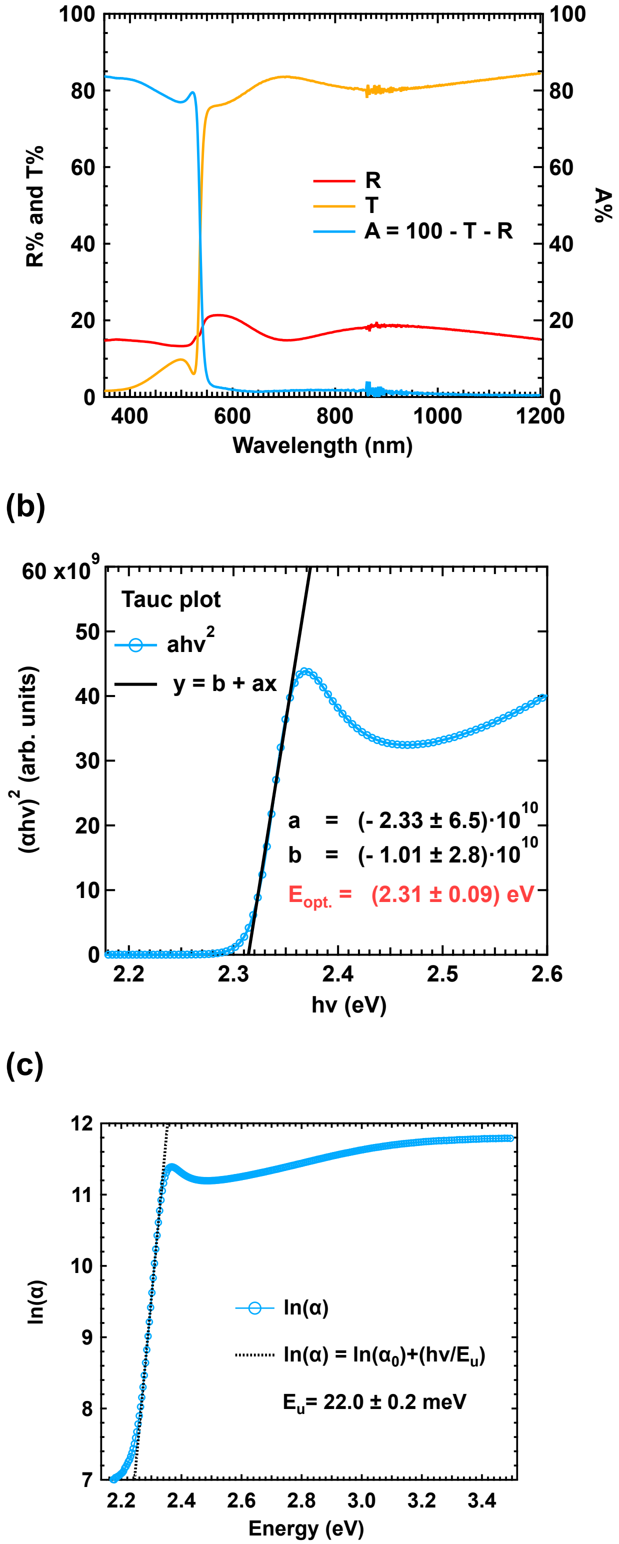

Figure S1. (a) Transmittance, reflectance and calculated absorptance, (b) Tauc plot and (c) calculation of the Urbach energy.

Taking the logarithm of eq. (4) gives:

$$\ln(\alpha) = \ln(\alpha_0) + \frac{hv}{E_u} \quad (4)$$

From the linear part of the graph, a value of E_u=22 meV is estimated.

S2. Fluorescence emission in $MAPbBr_3$

To obtain information on the nature of the species participating in the PL emission of $MAPbBr_3$ the integrated PL was measured at different excitation densities, once increasing and once decreasing, as shown in Figures S2 (a) and (b) respectively.

(a)

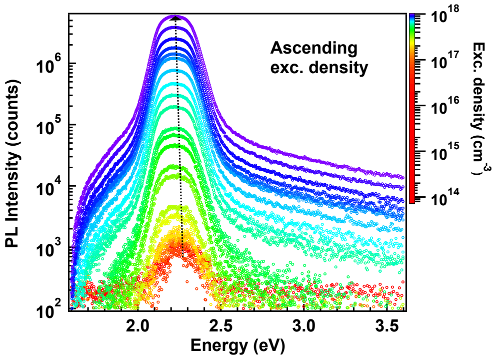

(b)

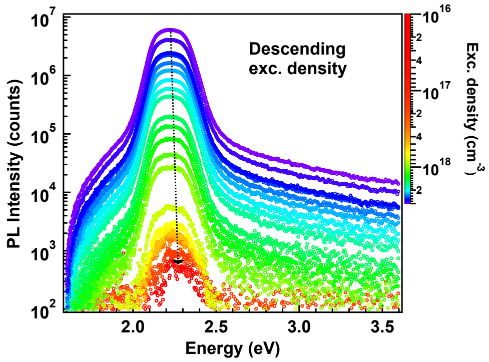

(c)

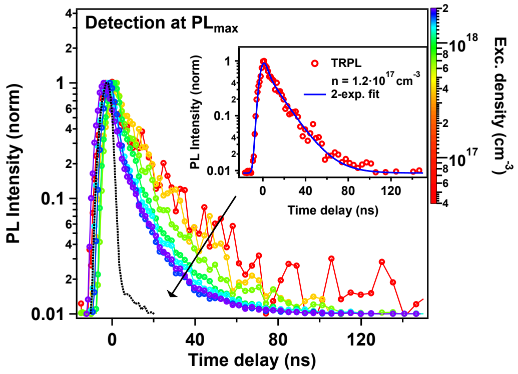

Figure S2 Integrated PL spectra obtained (a) with increasing and (b) with decreasing excitation carrier density. (c) TRPL dynamics detected at the PL maximum for different

excitation carrier densities. As an inset of Figure S2 (c), a two-exponential analysis of the PL dynamics at low excitation density is shown.

The excitation density was calculated by using the following equation:

$$n = (100 - T - R)\% \cdot \frac{E_{exc}}{h\nu \cdot \pi \cdot r^2 \cdot t} \quad (5)$$

where T is the transmittance, R the reflectance, E_{exc} the excitation energy per pulse, h the Planck's constant, ν the excitation frequency, r the radius of the laser beam, t the thickness of the film. In addition, the reflection from the chamber window that the sample was sealed in was also included for a more accurate estimation of the excitation carrier density.

Figure S2 (c) shows the TRPL dynamics detected at the maximum PL intensity, after excitation with 450 nm at different excitation carrier densities. The dynamics for the two lowest densities are identical, while for densities higher than $1.2 \cdot 10^{17}$ cm^{-3}, the dynamics start to become gradually faster. The dynamics that correspond to an excitation carrier density of $1.2 \cdot 10^{17}$ cm^{-3} were analyzed with a double exponential function convoluted with a gaussian function:

$$I_{PL} = y_0 + \frac{A}{2} \cdot e^{-\frac{t - t_0}{\tau_1}} \cdot e^{-\left(\frac{w}{2 \cdot \tau_1}\right)^2} + \frac{B}{2} \cdot e^{-\frac{t - t_0}{\tau_2}} \cdot e^{-\left(\frac{w}{2 \cdot \tau_2}\right)^2} \quad (6)$$

where y_0 stands for the background, *A* and *B* are the amplitudes, τ_1 and τ_2 the time constants corresponding to amplitudes *A* and *B* respectively, t_0 the time zero and *w* the pulse width. The values obtained from this procedure are *A* = 0.9, *B* = 0.1, τ_1 = 2 ns and τ_2 = 15 ns, giving an average PL lifetime of 3 ns, similar to the value reported in our previous study at a low excitation density.[3]

S3. Trap states probed with 480 nm excitation

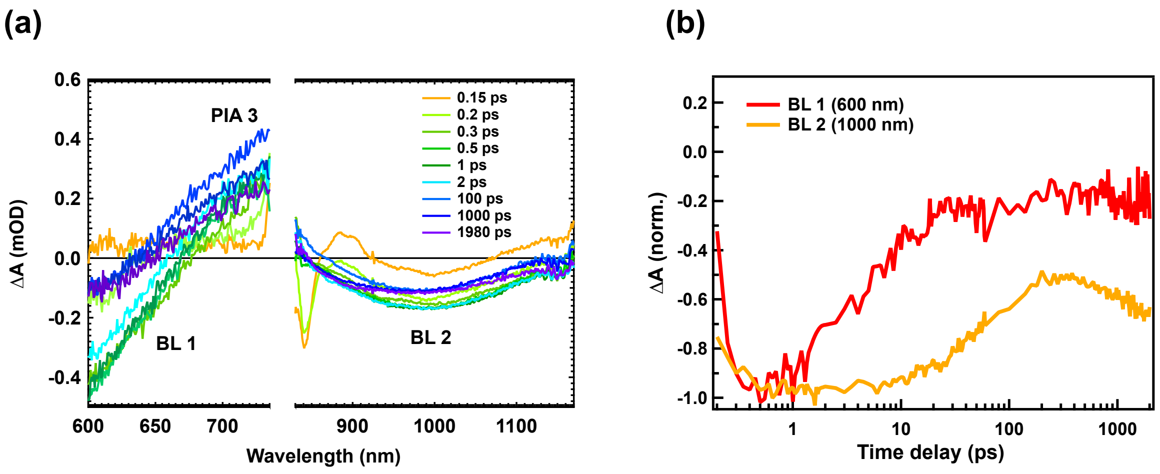

Figure S3 (a) Transient absorption spectra under 480 nm excitation detected in the infrared spectral region. (b) Normalized absorption change dynamics with 480 nm excitation at BL 1 (600 nm) and BL 2 (1000 nm).

At wavelengths between 565 nm – 660 nm, shown in Figure S3 (a), a negative signal (BL 1) reveals the presence of shallow trap states. Between 660 nm – 735 nm, a weak positive signal (PIA 3) starts appearing after 0.3 ps and remains present during the entire time window of the measurement. Detecting further in the IR, between 830 nm and 1150 nm, a weak negative signal (BL 2) is observed that reveals the presence of trap states deeper in the bandgap.

S4. Transient absorption spectra with 500 nm excitation

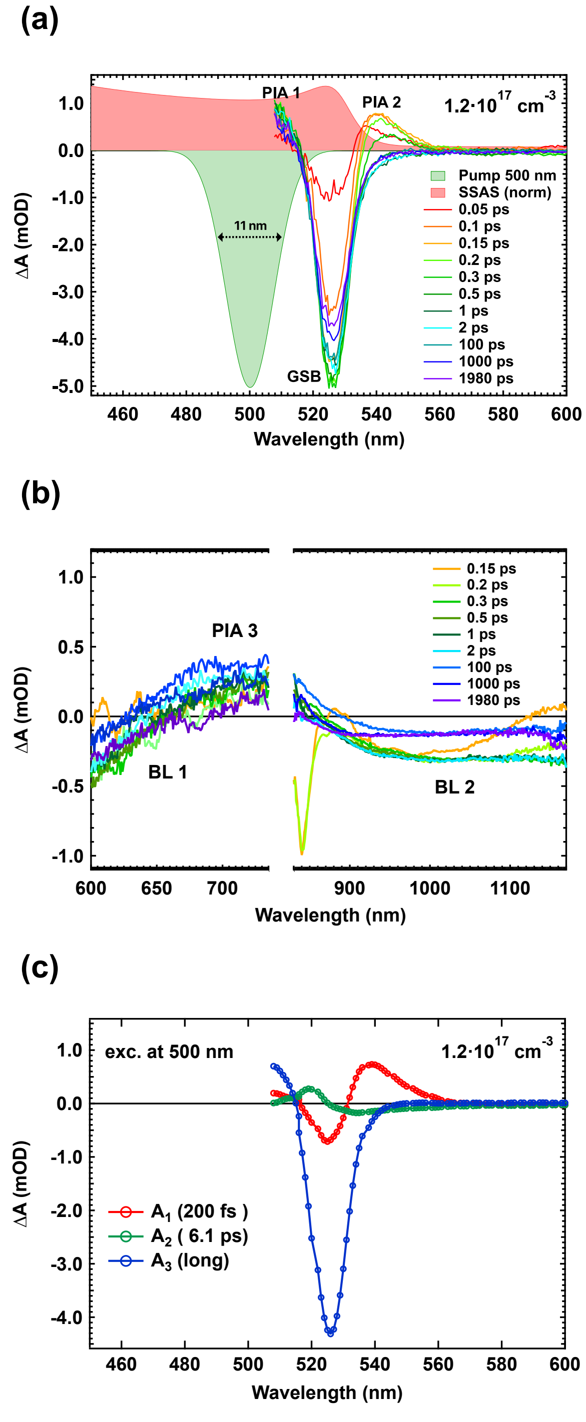

Figure S4 Transient absorption spectra detected (a) in the visible and (b) in the infrared spectral region at different time delays after excitation. The steady state absorption spectrum and the pump pulse that was used for the excitation are also shown. (c) Decay associated spectra of the $MAPbBr_3$ film excited at 500 nm.

The transient absorption spectra of the $MAPbBr_3$ film when excited with 500 nm pulses and detected in the visible range at selected time delays after photoexcitation are presented in Figure S4 (a). Similar spectral signatures as with 480 nm excitation are evidenced, showing that free carriers dominate the spectral signatures. PIA 1 is in this case not completely resolved because of its overlap with the 500 nm excitation pulses. Figure S4 (b) shows the TA spectra detected in the IR at different time delays. Similarly, to the 480 nm excitation, the weak negative signal (BL 2) is present when detecting deeper in the IR, is attributed to deep traps. Global analysis was performed, and the DAS are shown in Figure S4 (c). The DAS in the case of 500 nm excitation are very similar with the case of the 480 nm, and the derived time-constants can be attributed to the same recombination mechanisms.

S5. Trap states probed with 540 nm excitation

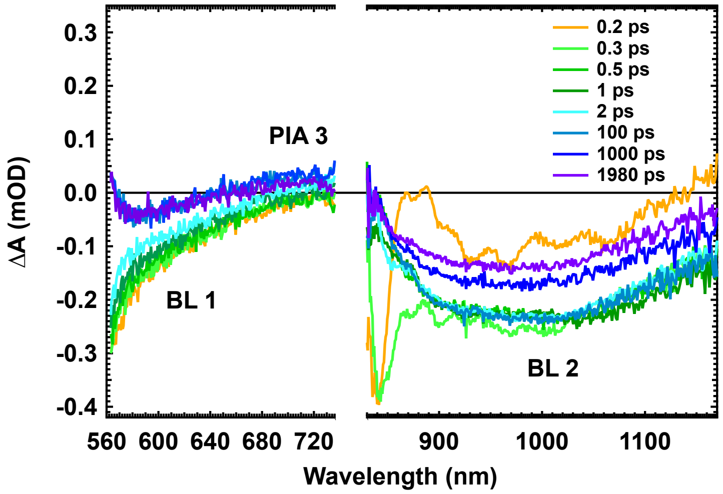

Figure S5 Transient absorption spectra at different time delays detected in the near-IR using 540 nm.

S6. Transient absorption spectra with 480 nm excitation at different densities

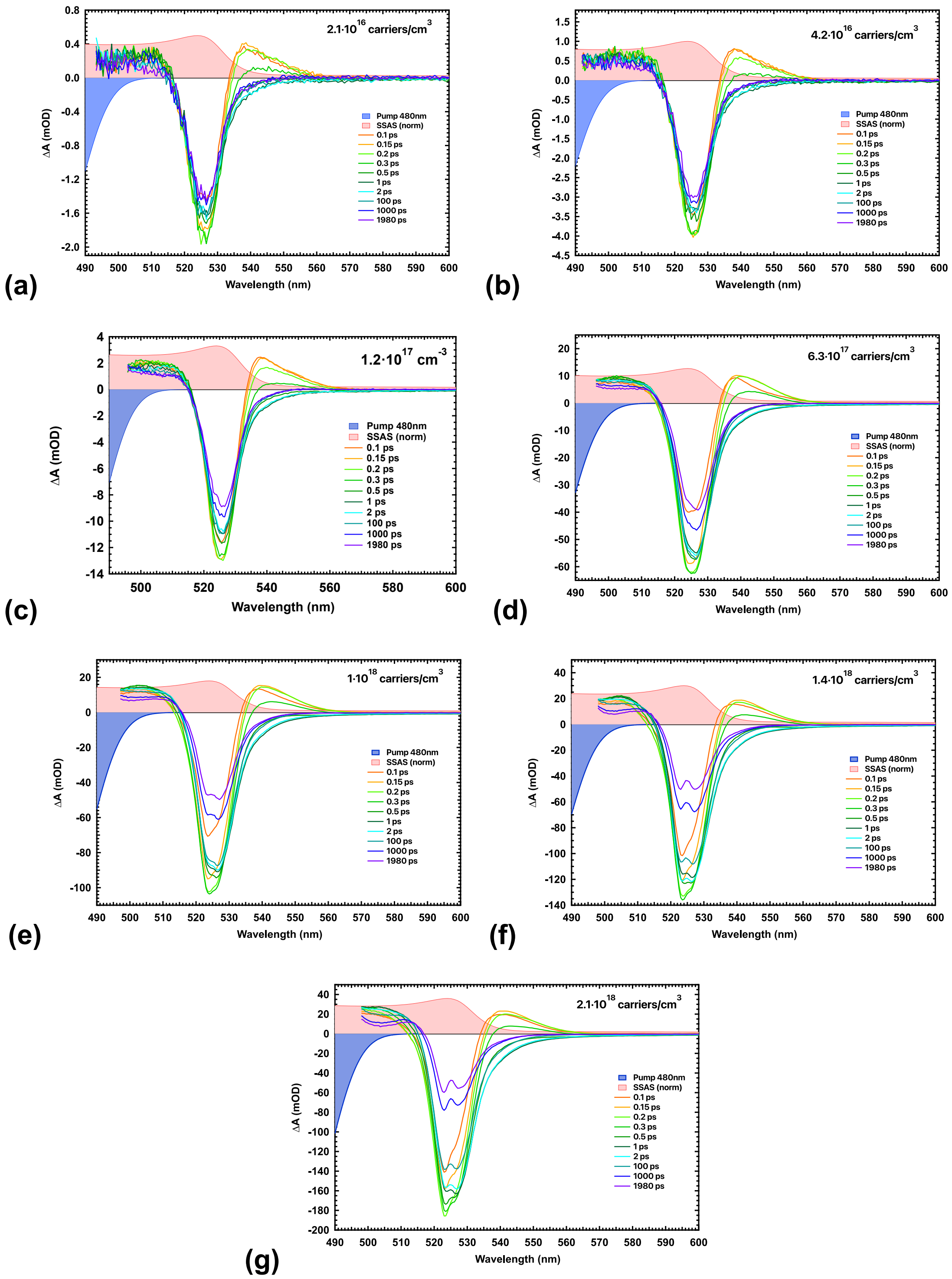

Figure S6 (a)-(g) Transient absorption spectra under different excitation densities with 480 nm excitation, detected in the visible region of the spectrum. The steady state absorption spectrum and the pump pulse that was used for the excitation are also shown.

S7. Transient absorption spectra with 540 nm excitation at different densities

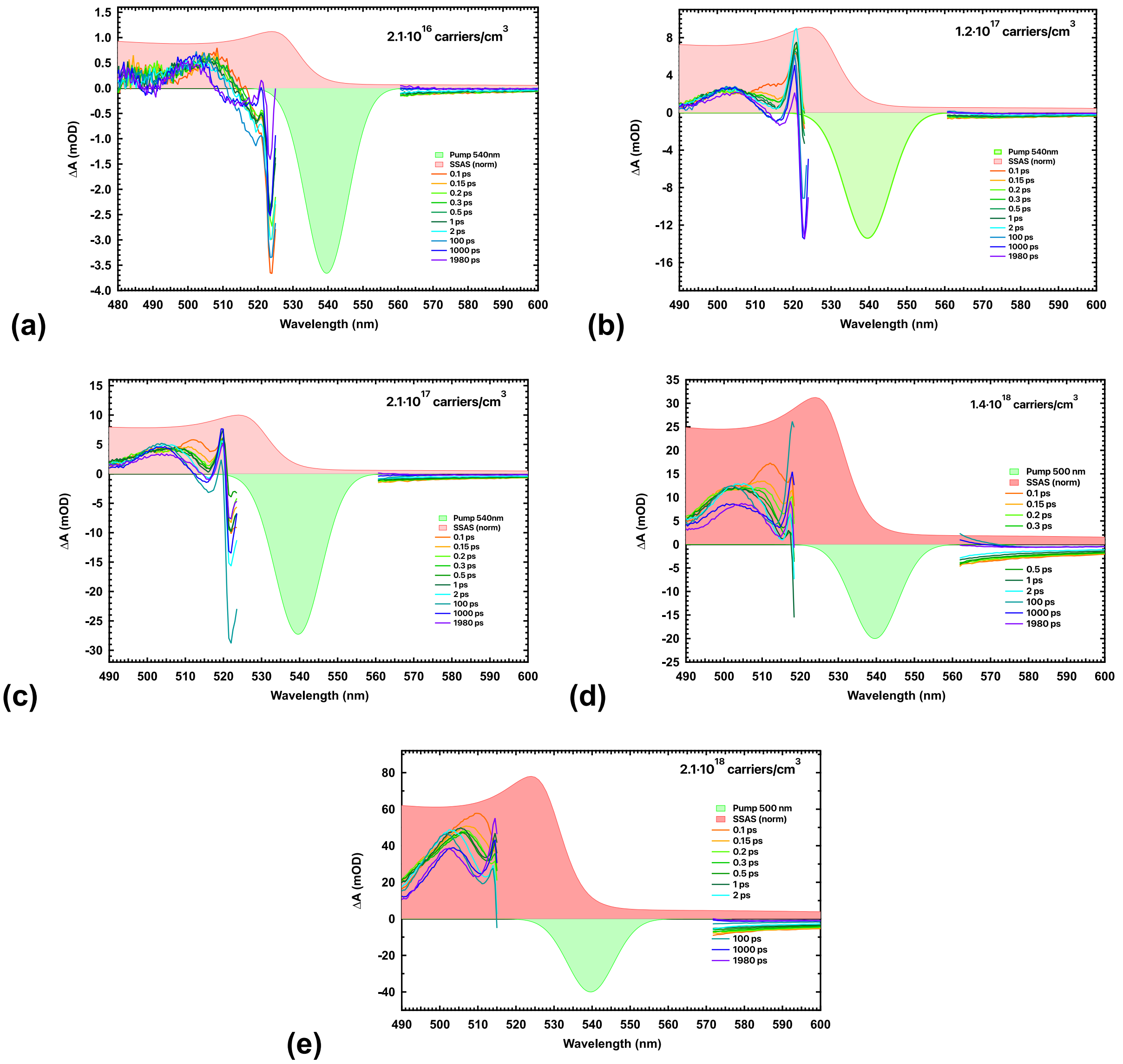

Figure S7 (a)-(e) Transient absorption spectra at different time delays under different excitation densities with 540 nm pulses, detected in the visible region of the spectrum. The steady state absorption spectrum and the pump pulse that was used for the excitation are also shown.

SI references

[1] J. M. Richter, M. Abdi-Jalebi, A. Sadhanala, M. Tabachnyk, J. P. H. Rivett, L. M. Pazos-Outón, K. C. Gödel, M. Price, F. Deschler, R. H. Friend, *Nat. Commun.* **2016**, *7*, 13941.

[2] V. Sarritzu, N. Sestu, D. Marongiu, X. Chang, Q. Wang, M. A. Loi, F. Quochi, M. Saba, A. Mura, G. Bongiovanni, *Adv. Opt. Mater.* **2017**, *6*, 1700839.

[3] N. Droseros, G. Longo, J. C. Brauer, M. Sessolo, H. J. Bolink, N. Banerji, *ACS Energy Lett.* **2018**, *3 (6)*, 1458.
